# A case report of interstitial keratitis and secondary glaucoma after cataract surgery that may be related to late congenital syphilis

**DOI:** 10.1186/s12886-023-02852-1

**Published:** 2023-04-28

**Authors:** Xingying Li, Yu Zhou, Huafeng Ma, Mingxing Wu

**Affiliations:** Department of Ophthalmology, The Second Affiliated Hospital of Chongqing Medical University, 400010 Chongqing, People’s Republic of China

**Keywords:** Secondary glaucoma, Interstitial keratitis, Cataract surgery, Congenital syphilis

## Abstract

**Background:**

The destruction of blood eye barrier and the administration of corticosteroid eyedrops after phacoemulsification surgery can lead to the replication of the local potential pathogens. With the rapid increase and popularization of cataract surgery, all kinds of rare postoperative complications have appeared. Here, we report a case of interstitial keratitis and secondary glaucoma after cataract surgery, which may be related to late congenital syphilis, which eventually led to blindness in the right eye. We hope that the timely report of this case will enable doctors to pay more attention to the possibility of potential pathogen replication after cataract surgery, and enable more patients to receive reasonable and effective treatment.

**Case presentation:**

A 63-year-old female was referred to our clinic for investigation with a 1-week history of moderate pain in the right eye and ipsilateral headache in January 2020. She had cataract surgery on her right eye two years ago and on her left eye one year ago. The intraocular pressure (IOP) in the right eye was 43.2 mmHg and that in the left eye was 28.5 mmHg. Her right eye underwent medication, trabeculectomy and finally was subjected to ciliary body photocoagulation to control the IOP. The IOP of the left eye was well controlled by regular use of eye drops. In addition to the elevated IOP, the inflammation of the anterior segment and corneal stroma was found. Before cataract surgery, bilateral corneal opacities was revealed, but after cataract surgery, interstitial keratitis in both eyes was gradually aggravated, during the follow-up period from 2019 to 2021. She informed us that she had suffered from decreased vision in both eyes and was diagnosed with bilateral keratitis and congenital syphilis at the age of 20. In 2018, the serologic test for syphilis was positive in blood (Chemiluminescence analysis (CLIA): + ; Toluidine red unheated serum test (TRUST): + , titer was 1:1). However, four tests for TRUST were negative in 2019 and 2020, so she was not treated for syphilis.

**Conclusion:**

This case of glaucoma and interstitial keratitis might be secondary to ocular inflammation caused by late congenital syphilis. The ocular inflammation and the activation of syphilis may be related to cataract surgery.

## Background

Syphilis is a chronic systemic disease caused by *treponema pallidum* infection. There are about 6 million new cases of syphilis worldwide every year [1]. The humans are the only source of syphilis, and the main routes of transmission are sexual transmission, iatrogenic transmission or vertical transmission. Syphilis is known as the "master of disguise". The common clinical manifestations of ocular syphilis include uveitis, retinal vasculitis and optic neuropathy. However, due to the changes in diagnosis strategies and evolving time, another kind of syphilis related ophthalmopathy is often ignored, that is interstitial keratitis related to congenital syphilis, which was more frequently reported earlier in the 1960s and 1970s. Here, we report a case of secondary glaucoma and interstitial keratitis 2 years after cataract surgery, which might be related to late congenital syphilis.

## Case presentation

A 63-year-old female was referred to our clinic for treatment with a 1-week history of moderate pain in the right eye and ipsilateral headache in January 2020.

**Fig. 1:**
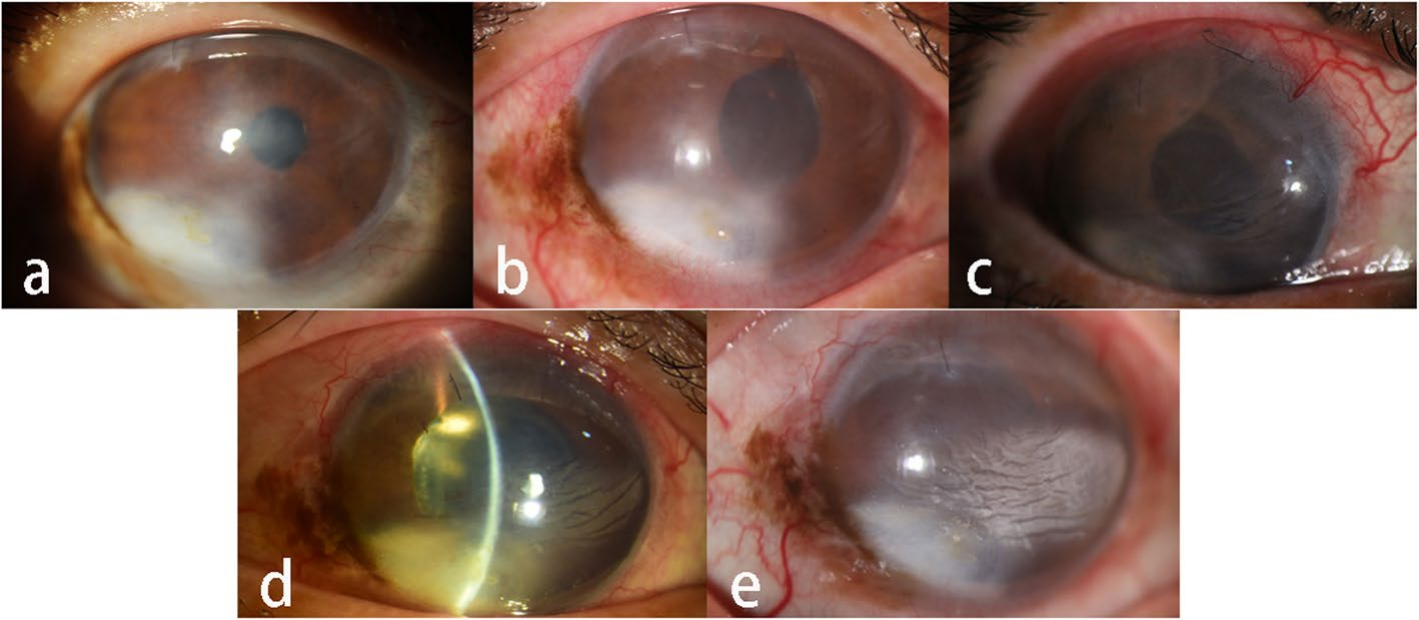
The corneal opacity and the ciliary hyperemia photos of the right eye at different stages: **a** IOP increased for the first time, 2 years after cataract phacoemulsification (January 2020). There was no obvious signs of ciliary congestion, the opaque area of the cornea was mainly located in lower part of the temporal area, and a small area of mild opaque could be seen in the nasal area. **b** There was obvious signs of the ciliary hyperemia and corneal transparency decreased significantly (March 2020). **c**, **d** and **e**. The sign of ciliary congestion was still obvious and the area of corneal opacity in the nasal part was progressively increased in July 2020, December 2020 and August 2021 respectively

**Fig. 2:**
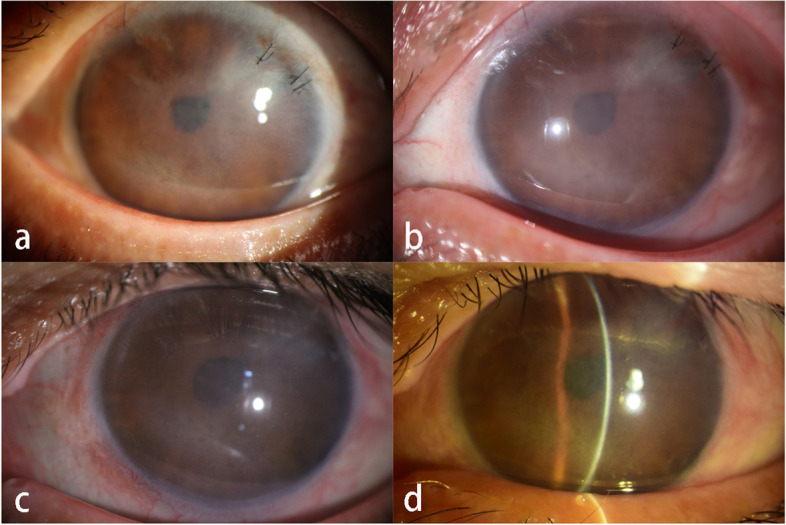
The corneal and ciliary hyperemia photos of the left eye at the different stages: **a** 10 months after cataract surgery (January 2020), there was mild corneal opacity, but no obvious signs of the ciliary congestion. **b** Nearly 13 months after the cataract surgery (March 2020), there was obvious signs of ciliary hyperemia and corneal opaque was worse as compared to 2 months ago; **c** and **d**. The sign of ciliary congestion was still obvious, and the corneal opaque did not appear to be significantly aggravated in July 2020 and December 2020 respectively

**Fig. 3:**
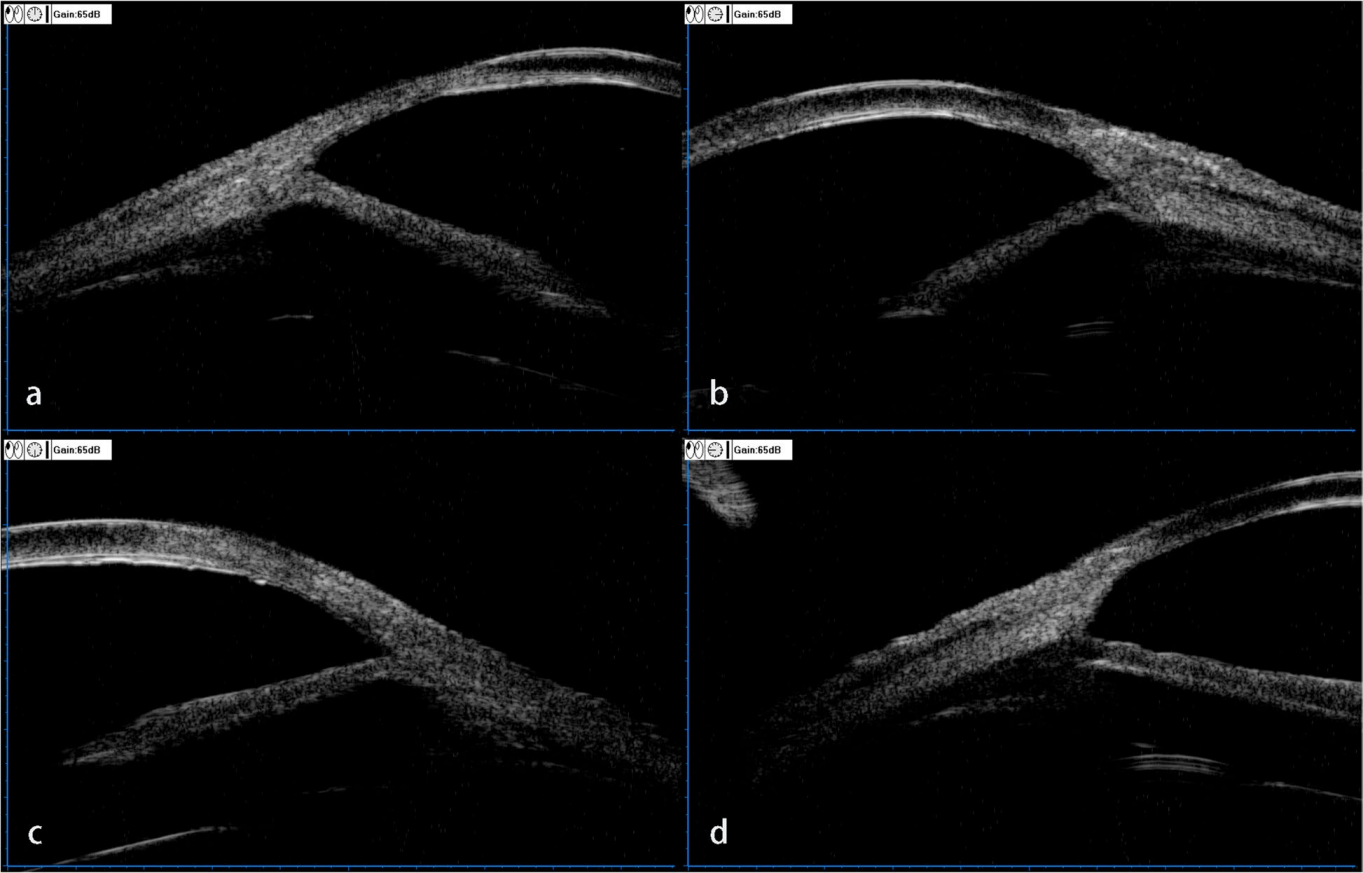
The ultrasound biomicroscopy (UBM) image of the right eye (January 2020), showed iris trabecular meshwork adhesion and anterior chamber angle closure at 12 o'clock, 3 o'clock, 6 o'clock and 9 o'clock respectively. **a**,**b**,**c**,**d** represent UBM image of anterior chamber angle at 12 o'clock, 3 o'clock, 6 o'clock and 9 o'clock, respectively

**Fig. 4:**
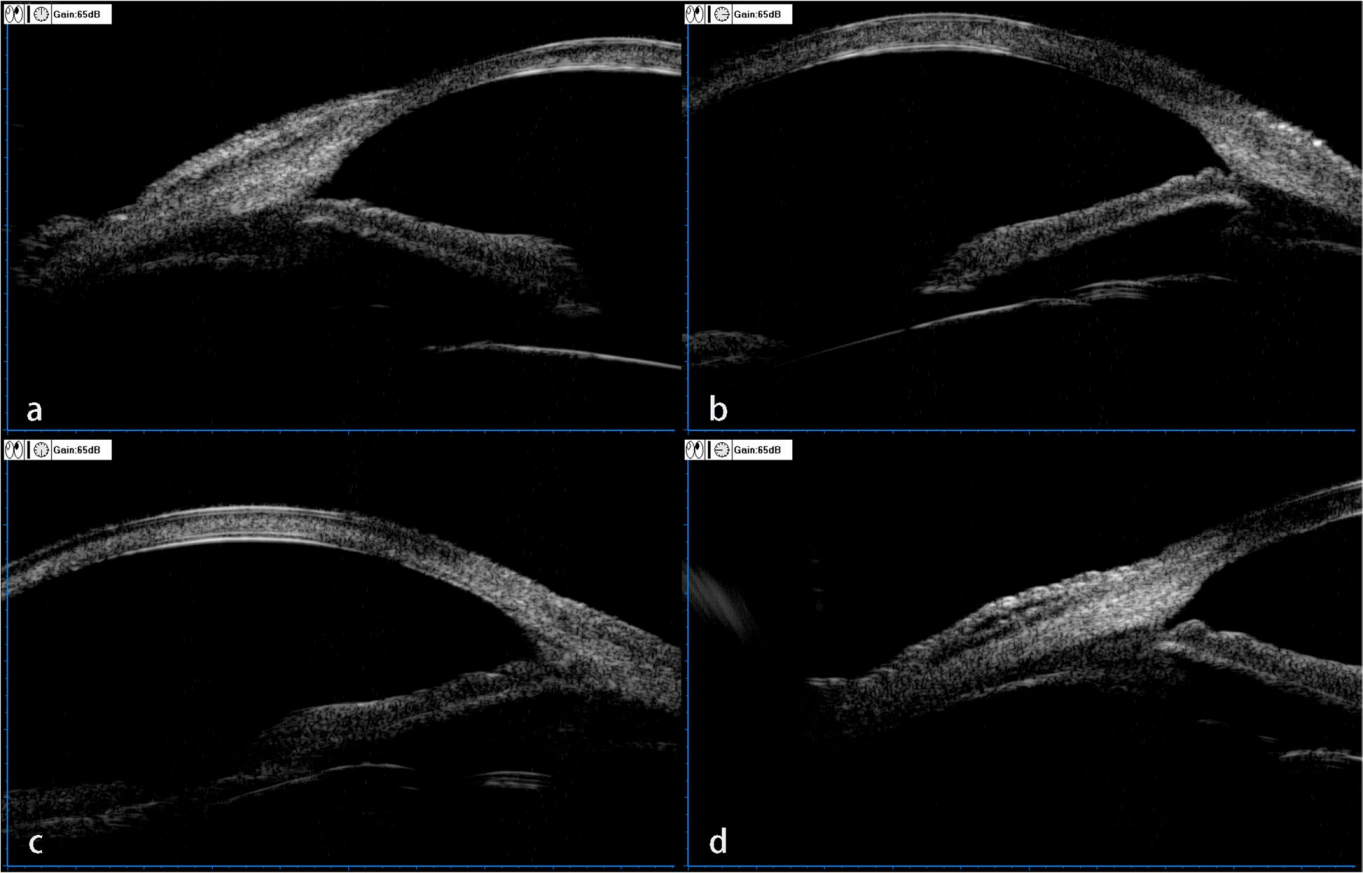
The ultrasound biomicroscopy (UBM) image of the left eye. There was no obvious detection of iris trabecular meshwork adhesion and angle closure. **a**, **b**, **c**, **d** represent UBM image of anterior chamber angle at 12 o'clock, 3 o'clock, 6 o'clock and 9 o'clock, respectively

She underwent cataract surgery on her right eye two years ago and on her left eye one year ago. Her uncorrected visual acuity (UCVA) in the right eye was LogMAR 1.70, and LogMAR 1.22 in the left eye. Her best corrected visual acuity (BCVA) in the right eye was LogMAR 0.70, and LogMAR 0.82 in the left eye. The intraocular pressure (IOP) in the right eye was 43.2 mmHg and that in the left eye was 28.5 mmHg. Corneal macula was found in the right eye (Fig. 1a), and the corneal nebula in the left eye (Fig. 2a). However, there were no obvious corneal edema in both eyes, no significant inflammatory cells and inflammatory reaction in the anterior chamber, no exudation and bleeding was found except choroidal atrophy. The anterior chamber angle of the right eye was closed due to at least 300 degrees of irido-trabecular contact (Fig. 3). For the left eye, the anterior chamber angle was closed about 30 degrees due to irido-trabecular contact (Fig. 4), and a large amount of the pigment covered the anterior chamber angle.

We considered that the increase of IOP could be possibly caused by ocular inflammation, but what contributed the inflammation was not clear, so we prescribed eye drops for treatment: including tobramycin/dexamethasone and travoprost for both eyes, carteolol and brimonidine tartrate for the right eye. At first, the IOP for both eyes was well controlled, however, three months later, the IOP of the right eye was uncontrolled and increased to 48.4 mmHg, so the patient underwent ciliary body photocoagulation of his right eye after informed consent. The retinal and choroid were the same as before, and no obvious inflammatory changes were noticed. Unfortunately, four months later, the IOP of the right eye was out of control and increased to 45.5 mmHg again. Finally, the right eye accepted ciliary body photocoagulation. Additionally, it was found that bilateral corneal opacities gradually aggravated after cataract surgery., and both eyes showed ciliary hyperemia, especially the right eye (Figs. 1b-d and 2b-d).

When we discussed the condition with patient, she told us that the visual acuity of her both eyes had been poor since she was 20 years old. At that time, she suffered from ocular inflammation due to late congenital syphilis for which she received penicillin as treatment. This important information made us to consider that the occurrence of Interstitial keratitis and secondary glaucoma might be related to syphilis, so we carried out her syphilis serological results. In 2018, she received cataract surgery on her right eye in other hospital. Before this surgery, the serologic test for syphilis was positive in blood (Chemiluminescence analysis (CLIA): + ; Toluidine red unheated serum test (TRUST): + , titer was 1:1). In 2019, she received cataract surgery on her left eye in our hospital, enzyme-linked immunosorbent assay (TP-ELISA) was positive, but TRUST was negative. At that time, we rashly diagnosed her as suffering from inactive syphilis, and thus missed the past diagnosis of congenital syphilis. In June 2020 and August 2021, the serological test results of TP-ELISA were positive, and the results of TRUST were negative. After consultation, the dermatologists believed that the patient had no syphilis active lesions all over the body, and hence she was not administered penicillin treatment.

From January 2020 to August 2021, when there was ciliary hyperemia, corticosteroid eye drops was used to control the excessive inflammation, at a frequency of 4 times/day progressively reduced every week for a total duration of 2 months. Unfortunately, in August 2021, the IOP of her right eye increased to 45 mmHg again, the UCVA of her right eye had fallen to counting fingers/40 cm and could not be rectified, and the corneal stroma was severely opaque and formed corneal leukoplakia. The patient refused further serological examination and aqueous humor test for syphilis, so her right eye was given ciliary body photocoagulation again. At present, the cornea of the right eye has become seriously opaque (Fig. 1e), the IOP of her right eye is controlled at about 11 mmHg, whereas the IOP of her left eye has remained normal.

## Discussion and conclusions

The initial diagnosis of this patient was difficult and even might be inaccurate. This is primarily because the previous diagnosis and treatment of congenital syphilis and keratitis were her own recollection. However, the patient was diagnosed with binocular non-ulcerative interstitial keratitis and multiple serological tests for syphilis were positive. In addition, she firmly denied unsafe sexual behaviors, and her mother was a syphilis patient. Therefore, the diagnosis of congenital syphilitic interstitial keratitis may be more reasonable.

Non-ulcerative interstitial keratitis is also considered as the most common ophthalmic finding of Cogan’s Syndrome (CS) [2–4]. However, Cogan’s Syndrome (CS) is a rare clinical disease that been classified as a primary variable vessel vasculitis with systemic involvement in up to 80% of patients, and when diagnosing CS, syphilis serological positivity has to be excluded [5]. In addition, the typical clinical presentation of CS are vestibuloauditory symptoms and non-syphilitic interstitial keratitis [5]. The patient serological tests for syphilis were found to be positive without vestibuloauditory symptoms and thus the diagnosis of CS was excluded.

Interstitial keratitis is the most common ocular manifestation of congenital syphilis that generally appears between 5 and 20 years of age [6–8]. It has been reported that in the interstitial keratitis associated with syphilis 90% were congenital syphilis, whereas only 10% were acquired [9–11]. Congenital syphilitic interstitial keratitis is typically a nonulcerative and nonsuppurative corneal inflammation which can be localized or diffused [12–14], and there might be an associated iridocyclitis with or without keratic precipitate [15]. Furthermore, Interstitial keratitis is a more or less vascularized inflammation of the corneal stroma. Untreated, the corneal inflammatory process may slowly regress, leaving a scarred cornea with narrowed stromal vessels, called “ghost” vessels, that may cause severe poor vision.

Tsukahara pointed out that the incidences of secondary glaucoma due to congenital syphilitic interstitial keratitis occupies 20% of the secondary glaucoma cases [16]. Meanwhile, congenital syphilitic interstitial keratitis usually be complicated with iridocyclitis, thereby resulting in trabecular pigmentation and anterior iris synechia, which eventually leads to secondary glaucoma [6]. The time interval between active keratitis and onset of the glaucoma range of from 20 to 30 years [16]. The onset time of secondary glaucoma in this patient coincided with this interval.

The diagnosis and treatment of this patient provided us with valuable experience. First, we were not able to identify the real cause of corneal opacity in detail during eye examination and just regarded the corneal opacity as a stable lesion caused by previous keratitis. This is because the patient did not reveal her initial history of congenital syphilis when she first visited our hospital for cataract surgery of the left eye and failed to inform us that her syphilis serology test was positive when she underwent cataract surgery in the right eye in other hospital a year ago. In addition, interstitial keratitis of left eye gradually progressed after cataract surgery, which was controlled corticosteroid eye drops. We suspect that cataract surgery probably induce the activation of treponema pallidum, as Yeung SN et al. reported that a patient with latent syphilis only developed syphilitic ophthalmopathy after cataract surgery, manifested as mild anterior segment inflammation [17]. Second, although we used corticosteroid eyedrops for the treatment of ocular inflammation, the corneal lesions in the right eye were still progressive, and no alternative strategies were employed to control the aggravation of corneal lesions in the right eye. Thus, after analyzing the literature, some immunosuppressants such as tacrolimus eyedrops were found to be effective in the management of corneal stromal inflammation [18].

However, some authors have reported that when there was only corneal stromal inflammation, penicillin treatment was not necessary [19]. In our case, there was no systemic penicillin treatment administered after consultation with dermatology department, as the corneal lesion in the patient's right eye was progressive, and the corneal stromal inflammation had not been effectively controlled. In addition to immunosuppressive drugs, it is not clear if penicillin treatment could be helpful, as in addition to the corneal stroma inflammation, severe iridocyclitis was also observed. The serological test result of TRUST was negative and hence maybe we could consider the detection of treponema pallidum in aqueous humor. If the test result is positive, penicillin treatment might be helpful to control the progression of the disease.

Finally, it is worth noting that in addition to paying attention to corneal stroma inflammation and intraocular pressure, we also need to consider the presence of other syphilis related diseases in sclera, optic nerve, retina and choroid, because syphilis is a master of camouflage, which can cause diseases in any part of the eye.

When interstitial keratitis occurs, we should carefully consider the possibility of syphilis infection, and cataract phacoemulsification probably might lead to the recurrence of syphilis infection.

## Data Availability

All the data supporting our findings are contained within the manuscript. The datasets used and/or analyzed during the current study available from the corresponding author on reasonable request.

## Abbreviations

IOP: Intraocular pressure
UCVA: Uncorrected visual acuity
BCVA: Best corrected visual acuity
TRUST: Toluidine red unheated serum test
TP-ELISA: Treponema pallidum enzyme-linked immunosorbent assay
CS: Cogan’s Syndrome
CLIA: Chemiluminescence analysis
